# Serum C-X-C Chemokine Ligand 1 Levels in Patients with Systemic Sclerosis: Relationship of Clinical and Laboratory Observations to Anti-CD20 Monoclonal Antibody Administration

**DOI:** 10.3390/life12050646

**Published:** 2022-04-27

**Authors:** Ruriko Kawanabe, Ayumi Yoshizaki, Kazuki M. Matsuda, Hirohito Kotani, Teruyoshi Hisamoto, Yuta Norimatsu, Ai Kuzumi, Takemichi Fukasawa, Satoshi Ebata, Asako Yoshizaki-Ogawa, Shinichi Sato

**Affiliations:** Department of Dermatology, Faculty of Medicines, The University of Tokyo, Bunkyo-Ku, Tokyo 113-8654, Japan; kawanaberuriko@gmail.com (R.K.); mkazuki.kom@gmail.com (K.M.M.); d10sm038@gmail.com (H.K.); hisamotot-der@h.u-tokyo.ac.jp (T.H.); norimanorima@gmail.com (Y.N.); kuzumia-der@h.u-tokyo.ac.jp (A.K.); takemichi.giraffe@gmail.com (T.F.); ebatas-der@h.u-tokyo.ac.jp (S.E.); asako56planetes730@yahoo.co.jp (A.Y.-O.); shinsato2020@gmail.com (S.S.)

**Keywords:** CXCL1, systemic sclerosis, fibrosis, intestinal lung disease, anti-CD20 antibody

## Abstract

**Objectives:** To determine whether C-X-C chemokine ligand 1 (CXCL1), which is a potent neutrophil chemoattractant and activator that plays important role in inflammation, is elevated in patients with systemic sclerosis (SSc) and whether it is associated with the clinical features and disease activity of patients with SSc. In addition, to determine whether the changes in serum CXCL1 levels before and after treatment correlate with changes in disease activity in SSc patients who received an anti-CD20 monoclonal antibody drug. **Patients and method:** We examined patient serum collected in the DesiReS trial, which was a double-blind, parallel-group, randomized, placebo-controlled, multicenter, phase II clinical trial. In the trial, patients were randomly allocated to the drug or placebo group and received 375 mg/m^2^ of an anti-CD20 antibody, rituximab, or placebo once a week for four weeks. We obtained serum samples from 47 patients administered at our hospital, including 3 males and 44 females, the median age of 48 years, range 27–71 years, with 42 diffuse cutaneous SSc and 5 with limited cutaneous SSc. Serum CXCL1 levels were measured using multiplex immunoassay in patient serum before and 24 weeks after administration and also in serum from 33 healthy controls. **Results:** Serum CXCL1 levels were significantly higher in SSc patients (mean 25.70 ng/mL; 95% confidence interval (CI) 18.35–33.05 ng/mL) than in the healthy controls (15.61 ng/mL; 95% CI 9.73–21.51 ng/mL). In addition, SSc patients with elevated CXCL1 levels had a significantly higher percentage of area occupied with interstitial shadows (*p* < 0.05), increased serum levels of surfactant protein (SP)-A (*p* < 0.05), SP-D (*p* < 0.05), Krebs von den Lungen-6 (*p* < 0.01), and C-reactive protein (*p* < 0.05) compared to those with normal levels. Furthermore, defining Δ as the value after rituximab administration minus the value before rituximab administration, baseline serum CXCL1 levels correlated with Δ percent predicted diffusing capacity for carbon monoxide (*p* < 0.01). In addition, ΔCXCL1 correlated with ΔSP-A (*p* < 0.05). Similarly, serum CXCL1 levels after rituximab administration correlated with percent predicted forced vital capacity (*p* < 0.05) and serum SP-D levels (*p* < 0.05) after rituximab. **Conclusions:** Our results suggest that serum CXCL1 is associated with the disease activity of SSc-ILD, and high serum CXCL1 levels are one of the predictors of improvement in SSc-ILD with rituximab.

## 1. Introduction

Systemic sclerosis (SSc) is a connective tissue disease characterized by tissue fibrosis and vasculopathy in various organs on a background of inflammation caused by autoimmune abnormalities [1]. Although the pathogenesis of SSc remains unclear, three major abnormalities, including abnormal immune cell activation, collagen accumulation, and vascular damage, are considered as a triad of disease conviction [2,3,4]. Collagen accumulation crucially causes fibrosis of several organs, such as skin, lungs, heart, and intestine. Vascular damage mainly induces Raynaud’s phenomenon, digital ulcers/gangrene, scleroderma renal crisis, and pulmonary hypertension. The most common cause of death with SSc is an SSc-associated interstitial lung disease (ILD), affecting 35–52% of patients [5].

It has been shown that B-cell abnormalities, particularly persistent abnormal activation of memory B cells, are important in the development of SSc [6], suggesting that B-cell-targeted therapy may be effective in SSc. Rituximab is an anti-CD20 monoclonal antibody that deletes B cells in the peripheral blood, and indeed, several studies have shown that rituximab is safe and effective against skin sclerosis and ILD in patients with SSc [7,8,9]. Recently, our investigator-initiated clinical trial (DesiReS) also demonstrated the efficacy and safety of rituximab for SSc, which has led to the regulatory approval of rituximab for the treatment of SSc in Japan [10]. At the same time, there are several safety problems involved in rituximab administration. A short-term problem is infusion reactions, which can cause fever, headache, chills, rash, and nausea within 24 h of administration. The medium-to long-term problem is infection; in a systematic review of rituximab administration for SSc-ILD, infections occurred in 13.5% of 112 SSc patients. Of these, respiratory tract infections were the most common (12 patients), followed by herpes zoster virus reactions (3 patients), urinary tract infections (2 patients), and hepatitis B virus reactivation (1 patient) [11]. In fact, we also experienced a case of acute exacerbation of interstitial pneumonia, probably due to infection after rituximab administration [12]. Because SSc patients with severe ILD have reduced lung capacity, even non-severe lung damage due to infection can be fatal. Therefore, it is important to predict which patients are suitable for treatment with anti-CD20 monoclonal antibody, especially for patients with SSc-ILD. However, there are still no clear indicators of which patients are suitable for rituximab treatment.

The chemokine C-X-C chemokine ligand (CXCL) 1, also known as growth-related oncogene-alpha, is a potent neutrophil chemoattractant and activator. CXCL1 was initially isolated and characterized based on its growth-stimulating activity against malignant melanoma cells and has been shown to associate with atherosclerosis, angiogenesis, and many chronic inflammations [13,14]. Serum CXCL1 levels have been shown to increase in several autoimmune diseases. In systemic lupus erythematosus, serum CXCL1 levels are elevated, suggesting an association with disease activity [15]. Previous study has also suggested that serum CXCL1 levels are elevated in SSc [16]. However, it is not fully clear whether CXCL1 is associated with SSc disease activity or treatment responsiveness.

The purpose of our study is to reevaluate whether serum CXCL1 levels are elevated and associated with disease activity in SSc patients and to determine whether serum CXCL1 is associated with response to rituximab treatment in SSc patients.

## 2. Experimental Section

### 2.1. Serum Sample from SSc Patient and Healthy Controls

This study was conducted using patient serum samples collected in the DesiReS trial, which was a double-blind, parallel-group, randomized, placebo-controlled, multicenter, phase II clinical trial [10]. Of the 56 patients who participated in the trial, we obtained serum samples from 47 patients administered at our hospital. Patients included 3 males and 44 females, the median age of 48 years, range 27–71 years, including 16 (34%) patients with no history of corticosteroid or immunosuppressive therapy, classified according to LeRoy’s classification rule into 42 (89.3%) with diffuse cutaneous SSc (dcSSc) and 5 (10.6%) with limited cutaneous SSc (lcSSc); mean disease duration were 96.1 months (standard deviation; 78.0 months). As a disease onset, Raynaud’s phenomenon was observed in 29 patients (61.7%), skin sclerosis or puffy fingers in 24 patients (51.1%), arthralgia in 2 patients (4.26%), and reflux esophagitis in 1 patient (2.13%). We collected the serum samples twice, before and 24 weeks after rituximab or placebo administration. Additionally, we obtained serum samples from 33 healthy controls (5 males and 28 females, median age 46 years, range 18–78 years). In this study, patients were randomly allocated to the drug or placebo group and received 375 mg/m^2^ of rituximab or placebo once a week for four weeks. Twenty-three were assigned to the rituximab group and twenty-four to the placebo group of the 47 SSc patients. Inclusion criteria were: meeting the American College of Rheumatology and European League Against Rheumatism criteria [17], a modified Rodnan Skin Score (mRSS) of at least 10, and expected survival of at least 6 months. Exclusion criteria were: the presence of pulmonary hypertension or serious disease complications associated with SSc, vital capacity <60%, administration of corticosteroids >10 mg per day within 2 weeks prior to sample collection, antifibrotic or immunosuppressive agents 4 weeks prior to sample collection, and cyclophosphamide in the past 2 years. The trial was conducted in accordance with the Declaration of Helsinki and the International Conference on Harmonization Good Clinical Practice guidelines. Written informed consent was obtained from all the patients and healthy controls. The study was also approved by the ethical committee of The University of Tokyo Hospital (No. 0695). Fresh venous blood samples were centrifuged shortly after clot formation. All samples were stored at −80 °C prior to use. 

### 2.2. Measurement of Serum CXCL1 Levels

Serum CXCL1 levels were examined using a commercially available multiplex immunoassay (MILLIPLEX MAP Human Cytokine/Chemokine/Growth factor Panel A. Millipore, Billerica, MA, USA) according to the manufacturer’s instructions. The measurements were outsourced to an inspection company.

### 2.3. Clinical Assessment of Patient

Because the study used data from a clinical trial, clinical data on SSc patients, including mRSS, blood tests, respiratory function tests, and chest computed tomography scans, were collected prospectively. Data on disease onset, disease duration, clinical features other than mRSS, and medications were collected by retrospective review of medical records. Disease duration was defined as the interval between the appearance of definite symptoms of SSc other than Raynaud’s phenomenon and obtaining serum samples. The severity of skin sclerosis was defined by the mRSS, which was measured as a total score of skin sclerosis scored at 17 sites [18]. The degree of skin sclerosis at each site was expressed at a score of 0–3 points. Cutaneous symptoms such as Raynaud’s phenomenon, nail fold breeding, telangiectasia, pitting scars, and skin ulcers were considered present if they were observed at least once during the disease period. Reflux esophagitis was defined as confirmed by the most recent gastric endoscopy, so patients who were well-maintained by medication were excluded. The area occupied by interstitial shadows in the lung was defined as the average of the percentage of interstitial shadows in each section of the lung cut at five levels of height on chest computed tomography.

### 2.4. Statistical Analysis

We used the Kruskal–Wallis test for multiple comparisons, Mann–Whitney’s U-test for two-group comparisons, Wilcoxson signed rank test for paired comparison, and Fisher’s exact probability test for comparison of frequency. Spearman’s rank correlation analysis was used to examine the relationship between two continuous variables. *p* < 0.05 was considered statistically significant.

## 3. Result

### 3.1. Serum CXCL1 Levels in SSc Patients and Healthy Controls

**Figure 1:**
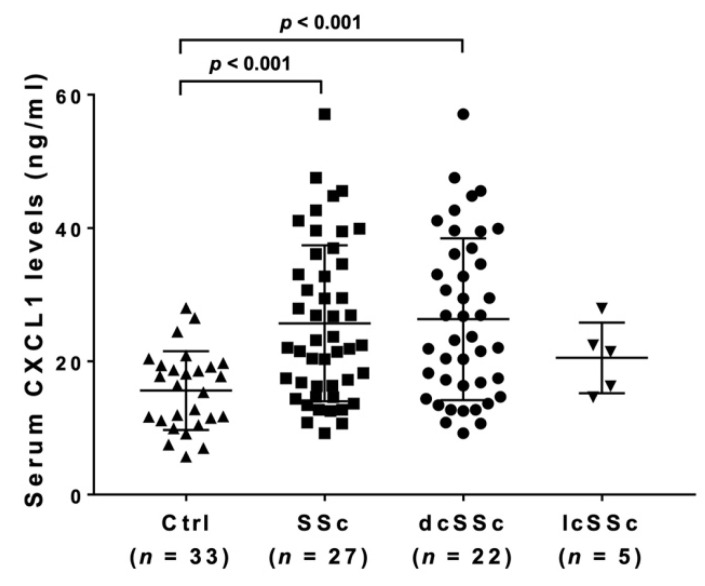
**Serum CXCL1 levels in SSc.** Serum CXCL1 levels were measured by a multiplex assay. The horizontal line in each column shows the mean. The Kruskal–Wallis test was conducted for multiple-group comparison. Ctrl, healthy controls.

Serum CXCL1 levels were detected in all samples of SSc patients while undetectable in 18% (6/33) of healthy controls. Even with the undetectable healthy controls as an exception, serum CXCL1 levels were significantly higher in SSc patients (mean 25.70 ng/mL; 95% confidence interval (CI) 18.35–33.05 ng/mL) than in the healthy controls (15.61 ng/mL; 95% CI 9.73–21.51 ng/mL; Figure 1). In subgroup analysis, serum CXCL1 levels were significantly higher in dcSSc (26.31 ng/mL; 95% CI 18.26–34.37 ng/mL) compared to healthy controls, but there was no significant difference between lcSSc (20.52 ng/mL; 95% CI 2.53–38.51 ng/mL) and healthy controls or dcSSc.

### 3.2. Clinical and Laboratory Features of SSc Patients with Elevated Serum CXCL1 Levels

**Figure 2:**
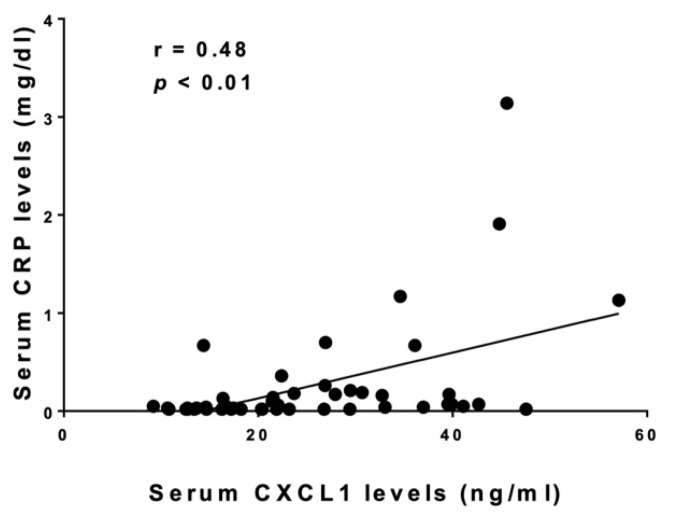
**The correlation between serum CXCL1 levels and serum CRP levels in SSc.** The solid line shows the regression line. Spearman’s rank correlation coefficient (r) was calculated for correlation analysis.

**Figure 3:**
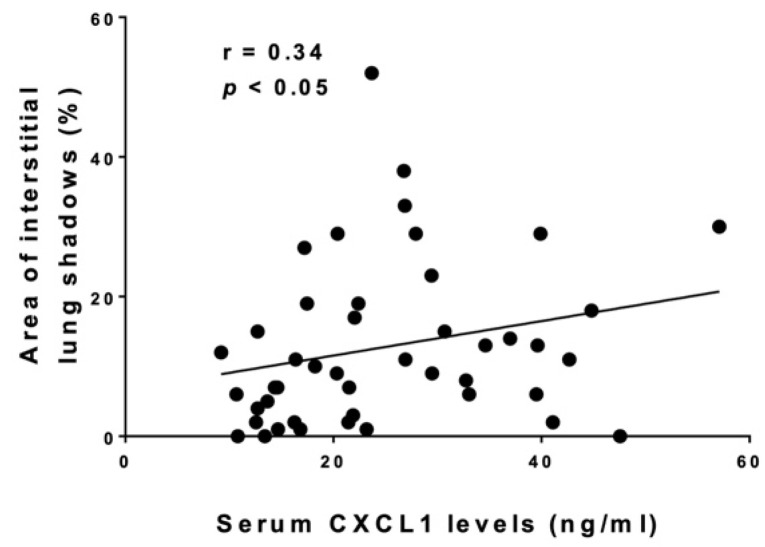
**The correlation between serum CXCL1 levels and area of interstitial lung shadows in SSc.** The solid line shows the regression line. Spearman’s rank correlation coefficient (r) was calculated for correlation analysis.

We compared clinical and laboratory features between SSc patients with elevated serum CXCL1 levels and those with normal levels (Table 1). The cut-off value was set at 21.51 ng/mL (the upper limit of the 95% confidence interval of serum CXCL1 levels in healthy controls). There were no significant differences in age, sex, clinical features, type of autoantibodies, present medications, percent predicted FVC, or diffusing capacity for carbon monoxide (DLco) between the two groups. On the other hand, patients with elevated CXCL1 levels had a significantly higher percentage of area occupied with interstitial shadows (*p* < 0.05), increased serum levels of SP-A (*p* < 0.05), SP-D (*p* < 0.05), Krebs von den Lungen (KL)-6 (*p* < 0.01), and C-reactive protein (CRP; *p* < 0.05) compared to those with normal levels. In addition, examining the correlation between serum CXCL1 levels and these clinical and laboratory findings, we found a significant correlation between serum levels of CXCL1 and CRP (r = 0.481, *p* < 0.01; Figure 2) and percentage of areas occupied with interstitial shadows of the lung (r = 0.339, *p* < 0.05; Figure 3) but not between CXCL1 and SP-A, SP-D, or KL-6 levels. Meanwhile, we examined the mean of serum CXCL1 levels by autoantibodies (Table 2). There were no significant differences between the three groups.

### 3.3. Changes in Serum CXCL1 Levels after Rituximab Administration

**Figure 4:**
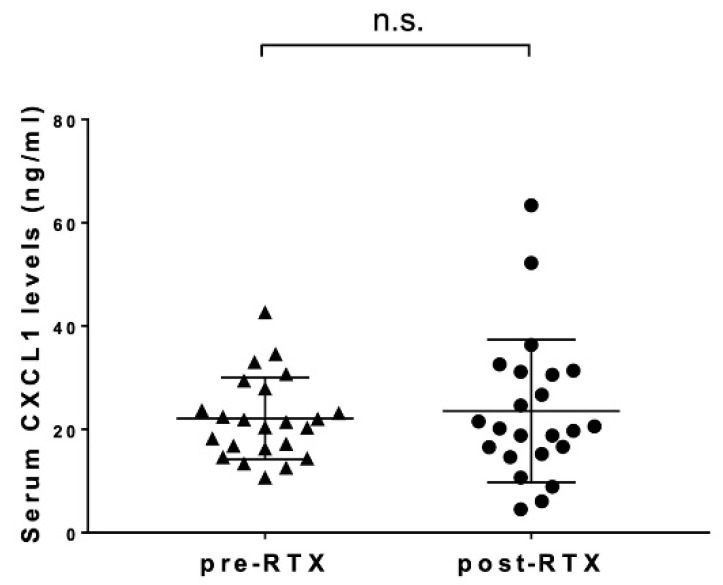
**The correlation of serum CXCL1 levels before and after rituximab administration.** The relationship between serum CXCL1 levels before rituximab administration (pre-RTX) and after rituximab administration (post-RTX) is shown. n.s.; no significance. Serum CXCL1 levels were measured by a multiplex assay. The horizontal line in each column shows the mean. Wilcoxson signed rank test was conducted for paired comparison.

Serum CXCL1 levels were compared before and 24 weeks after treatment in 23 SSc patients who received rituximab (Figure 4). Serum CXCL1 levels after rituximab administration (mean 23.53 ng/mL, 95% CI 13.91–33.15 ng/mL) were not significantly different from those before rituximab treatment (22.08 ng/mL, 95% CI 13.06–31.10 ng/mL). 

### 3.4. The Correlations between Serum CXCL1 Levels and Clinical Symptoms and Laboratory Findings during Rituximab Treatment

We examined the associations between serum CXCL1 levels and clinical symptoms and laboratory findings during rituximab administration by subtracting the pre-treatment value from the post-treatment value, expressing Δ (Table 3). As a result, baseline serum CXCL1 levels correlated with Δ%DLco (*p* < 0.01). Furthermore, there were correlations between ΔCXCL1 and ΔSP-A (*p* < 0.05) and between serum levels of CXCL1 and SP-D after rituximab administration (*p* < 0.05). In addition, serum CXCL1 levels after rituximab were negatively correlated with %FVC after rituximab administrations (*p* < 0.05).

## 4. Discussion

This study showed that serum CXCL1 was higher in SSc patients than in healthy controls. Furthermore, in SSc patients with elevated serum CXCL1 levels, serum markers of ILD and the percentage of areas occupied with interstitial shadows of the lung were significantly higher than in the normal group. In addition, serum CXCL1 levels correlated with serum CRP levels, which has been shown to correlate with disease activity, severity, decreased lung function, and shorter survival in the early stages of SSc within 3 years of onset [19] and percentage of areas occupied with interstitial shadows of the lung. These results suggest that serum CXCL1 levels are associated with disease activity of SSc-ILD and even prognosis in SSc patients. 

A previous study including 78 SSc patients with no exclusion criteria showed that serum CXCL1 levels in SSc patients were higher than in healthy controls. In addition, SSc patients with elevated serum CXCL1 levels showed an increased frequency of decreased % vital capacity, decreased % DLco, kidney involvement, presence of anti-topoisomerase I antibodies, and elevated serum IgG levels compared to patients with normal CXCL1 levels [16]. On the other hand, in this study, SSc patients with elevated serum CXCL1 levels were associated with active and severe pulmonary symptoms, as described above, but high serum CXCL1 levels were not significantly associated with the type of antibody, serum creatinine levels, or serum IgG levels. The first possible reason for this is that the sample size was smaller than in the previous study. In particular, the number of patients with anti-centromere antibodies was smaller because patients with mRSS greater than 10 were included in this study. Another reason is that patients with less severe organ damage were examined because of the exclusion criteria. CXCL1 is one of the most important chemokines, which is involved in the pathogenesis of many inflammatory diseases; CXCL1 is upregulated in inflammatory responses and induces angiogenesis and recruits neutrophils [20]. Whereas the utility of bronchoalveolar lavage (BAL) in the evaluation of SSc-ILD remains controversial, fractional analysis of BAL (FBAL), which is a technique that can analyze small airways and alveolar compartments separately, has proven informative in other ILDs [21]. Previous study has shown that in SSc-ILD, a higher percentage of neutrophils in FBAL-3, which contains alveolar components, is correlated with the development of end-stage ILD as well as mortality [22]. Other study has shown that CXCL1 levels in BALF closely correlate with the percentage of neutrophils in BALF. It has also been shown that in autoimmune interstitial pneumonia and idiopathic interstitial pneumonia, elevated plasma levels of CXCL1 are clinically associated with DLco, erythrocyte sedimentation rate, and lung parenchymal extension [23].

Based on the above, it is suggested that in SSc, elevated CXCL1 induces neutrophils in the alveoli, causing inflammation and exacerbating SSc-ILD. Furthermore, in a study examining the correlation between serum CXCL1 and clinical and laboratory findings after receiving an anti-CD20 monoclonal antibody drug, baseline serum CXCL1 correlated with Δ%DLco. In other words, the higher baseline serum CXCL1 levels, the greater the improvement in %DLco expected with rituximab, meaning that serum CXCL1 levels can be a predictor of improvement in lung function with rituximab. There were also correlations between ΔCXCL1 and ΔSP-A, between CXCL1 levels after rituximab and %FVC after rituximab, and between CXCL1 levels after rituximab and SP-D levels after rituximab, suggesting that serum CXCL1 levels are involved in SSc-ILD progression, and there are B-cell-related mechanisms there. 

Previous studies have shown that CXCL1 expression is increased by cytokines such as interleukin (IL) -1β, tumor necrosis factor-α [24,25], and IL-17 through NF-κB activation [26,27,28]. It has also been shown that CXCL1 is upregulated in salivary gland tissue in Sjögren’s syndrome and that stimulation with IL-6 increases the expression of CXCL1 and its receptor, C-X-C motif chemokine 2 [29]. IL-6 is a classic proinflammatory cytokine and is also considered to be an important protein in the immunopathogenesis of SSc. For example, several studies have shown that IL-6 levels in the skin, serum, and bronchoalveolar lavage fluid of SSc patients are elevated and play a role in promoting fibrosis by enhancing inflammation [30,31,32]. In immunohistochemistry, it has been shown that IL-6 is overexpressed in the endothelium and fibroblasts of SSc patients compared to normal skin [33]. Other study shows that dermal fibroblasts from SSc patients produce increased amounts of IL-6 compared to healthy control fibroblasts [34] and that B cells induce IL-6 secretion from lung fibroblasts. [35]. Recently, B-cell-activating factor (BAFF), an essential component of B-cell homeostasis and a potent B-cell survival factor associated with autoimmune disease, was shown to be increased in SSc patients compared to healthy controls [36], and the ability of B cells to produce IL-6 is significantly increased by BAFF stimulation [37]. In fact, in a study observing serum IL-6 levels in SSc patients after rituximab administration, patients had high levels of serum IL-6 at baseline, which decreased remarkably after 6 months following circulating B cells which, evaluated by flow-cytometry, were depleted [38]. 

From the above, it is suggested that the deletion of B cells reduces IL-6 that is produced and induced by B cells, resulting in a decrease in CXCL1, which is one of the reasons for the improvement in lung function and skin sclerosis after B-cell-depletion therapy with anti-CD20 monoclonal antibodies in SSc patients. At the same time, in this study, rituximab did not significantly decrease serum CXCL1 levels in SSc patients, meaning that various factors other than B cells may be related to the high serum CXCL1 levels in SSc. For example, T helper 17 cells, which produce IL-17, are proven to be involved in the pathogenesis of multiple autoimmune diseases, such as systemic lupus erythematosus [39], rheumatoid arthritis [40], and psoriasis [41], and some studies support an association with SSc [42,43], which may contribute to it.

There are several limitations to this study. First, the sample size was relatively small. In addition, because of several exclusion criteria, some groups of SSc patients were not studied, such as patients with very low pulmonary function or those with pulmonary hypertension. Furthermore, this study was limited to Japanese patients. However, because this study uses data from clinical trials, many of the data were collected prospectively. Therefore, the timing of the measurements is aligned, which is a strength.

Taken together, in this study, we showed that serum CXCL1 levels were involved in the pathogenesis of SSc-ILD and that high serum CXCL1 levels could be a predictor of SSc-ILD response to rituximab.

## Figures and Tables

**Table 1:** Clinical and laboratory findings of patients divided by elevated serum CXCL1 levels.

	Elevated CXCL1 Levels (*n* = 27)	Normal CXCL1 Levels (*n* = 20)
Age, years	47 (27–71)	50 (27–78)
Sex (male/female)	3/24	0/20
Clinical features		
dcSSc	25 (92.6%)	17 (85.0%)
lcSSc	2 (7.4%)	3 (15%)
mRSS	13 (10–28)	14 (10–31)
Raynauds phenomenon, %	25 (92.6%)	19 (95.0%)
Nail fold breeding, %	19 (70.4%)	12 (60.0%)
Telangiectasia, %	11 (40.7%)	6 (30.0%)
Pitting scars, %	10 (37.0%)	10 (50.0%)
Skin ulcer, %	12 (44.4%)	10 (50.0%)
Reflex oesophagitis, %	7 (25.9%)	5 (23.52%)
Autoantibodies		
Anti-topoisomerase I Ab, %	17 (63%)	10 (50%)
Anti-centromere Ab, %	5 (18.5%)	3 (15%)
Anti-RNA polymerase III Ab, %	2 (7.40%)	3 (15%)
Area occupied with interstitial shadows, % of lung fields.	13 * (1–52%)	**6.5 (1–29%)**
Laboratory findings		
%FVC, %	83.6 (72.2–108)	88.3 (63.2–124)
%DLco, %	78.8 (63.1–125)	88.75 (60.8–122)
SP-A, ng/mL	40.3 * (9.2–149)	29.8 (13.3–71.6)
SP-D, ng/mL	153 * (26.8–363)	120 (25.5–318)
KL-6, ng/mL	488 ** (84–3370)	**392 (141–2534)**
CRP, mg/dL	0.16 * (0.02–1.17)	0.03 (0.02–0.67)
IgG, mg/dL	1400 (766–2909)	1251 (703–1692)
BNP, pg/mL	25.7 (4–275)	15.3 (4–41.5)
Creatinin, mg/dL	0.61 (0.45–0.73)	0.62 (0.46–0.87)
Present medications		
Systemic corticosteroid use, %	17 (63%)	9 (45%)
Dose of systemic corticosteroid, mg/day	3.87 (3.52)	3.40 (4.03)
ERA and/or PDE-5 inhibitor use	18 (66.7%)	9 (45%)
Proton pump inhibitor use	24 (88.9%)	16 (45%)

Data are median (range) or *n* (%) or mean (SD) unless otherwise noted. *n*, number; mRSS, modified Rodnan total skin thickness score; BNP, brain natriuretic peptide; ERA, endothelin receptor antagonist; PDE-5, phosphodiesterase type 5. Statistical analysis was performed by Mann–Whitney’s U-test for continuous variables and Fisher’s exact probability test for comparison of frequency. * *p* < 0.05 or ** *p* < 0.01 vs. SSc patients with normal CXCL1 levels. ** *p* < 0.01 is shown in bold.

**Table 2:** Serum CXCL1 levels by autoantibody.

Autoantibody	*n*	Serum CXCL1 Levels
Anti-topoisomerase I Ab	27	23.2 (24.5–26.0)
Anti-centromere Ab	8	27.4 (26.2–31.6)
Anti-RNA polymerase III Ab	5	20.3 (16.9–21.5)

Data are mean (95% CI). *n*, number; Ab, antibody. Statistical analysis was carried out by Kruskal–Wallis test.

**Table 3:** Correlations between serum CXCL1 levels and clinical and laboratory parameters during rituximab administration.

Correlation	Strength of Correlation (r)
Pre-CXCL1 levels vs. Δclinical/laboratory data	
Pre-CXCL1 levels vs. ΔmRSS	0.163
Pre-CXCL1 levels vs. Δ%FVC	0.260
Pre-CXCL1 levels vs. Δ%DLco	0.531 **
Pre-CXCL1 levels vs. ΔSP-A	0.130
Pre-CXCL1 levels vs. ΔSP-D	0.018
Pre-CXCL1 levels vs. ΔKL-6	−0.177
Pre-CXCL1 levels vs ΔArea occupied with interstitial shadows	−0.305
ΔCXCL1 vs. Δclinical/laboratory data	
ΔCXCL1 vs. ΔmRSS	−0.222
ΔCXCL1 vs. Δ%FVC	−0.275
ΔCXCL1 vs. Δ%DLco	−0.122
ΔCXCL1 vs. ΔSP-A	−0.500 *
ΔCXCL1 vs. ΔSP-D	−0.116
ΔCXCL1 vs. ΔKL-6	0.211
ΔCXCL1 vs ΔArea occupied with interstitial shadows	−0.098
Post-CXCL1 levels post vs. post-clinical/laboratory data	
Post-CXCL1 levels vs. post-mRSS	0.145
Post-CXCL1 levels vs. post-%FVC	−0.511 *
Post-CXCL1 levels vs. post-%DLco	−0.379
Post-CXCL1 levels vs. post-SP-A levels	0.232
Post-CXCL1 levels vs. post-SP-D levels	0.498 *
Post-CXCL1 levels vs. post-KL-6 levels	0.178
Post-CXCL1 levels vs post-Area occupied with interstitial shadows	0.115

Pre, the values before rituximab administration; Post, the values after rituximab administration; Δ, the value after rituximab administration minus the value before rituximab administration. Values represent nonparametric correlations (Spearman’s r). * *p* < 0.05, ** *p* < 0.01.

## Data Availability

The data presented in this study are available on request from the corresponding author. The data are not publicly available due to ethical reasons.

## References

[1] LeRoy E.C., Black C., Fleischmajer R., Jablonska S., Krieg T., Medsger T.A., Rowell N., Wollheim F. (1988). Scleroderma (systemic sclerosis): Classification, subsets and pathogenesis. J. Rheumatol..

[2] Furst D.E., Clements P.J. (1997). Hypothesis for the pathogenesis of systemic sclerosis. J. Rheumatol. Suppl..

[3] Yoshizaki A., Yanaba K., Iwata Y., Komura K., Ogawa A., Muroi E., Ogawa F., Takenaka M., Shimizu K., Hasegawa M. (2011). Elevated serum interleukin-27 levels in patients with systemic sclerosis: Association with T cell, B cell, and fibroblast activation. Ann. Rheum. Dis..

[4] Yoshizaki A., Komura K., Iwata Y., Ogawa F., Hara T., Muroi E., Takenaka M., Shimizu K., Hasegawa M., Fujimoto M. (2009). Clinical significance of serum HMGB-1 and sRAGE levels in systemic sclerosis: Association with disease severity. J. Clin. Immunol..

[5] Bergamasco A., Hartmann N., Wallace L., Verpillat P. (2019). Epidemiology of systemic sclerosis and systemic sclerosis-associated interstitial lung disease. Clin. Epidemiol..

[6] Sato S., Fujimoto M., Hasegawa M., Takehara K., Tedder T.F. (2004). Altered B lymphocyte function induces systemic autoimmunity in systemic sclerosis. Mol. Immunol..

[7] Rubio-Rivas M., Royo C., Simeón C.P., Corbella X., Fonollosa V. (2014). Mortality and survival in systemic sclerosis: Systematic review and meta-analysis. Semin. Arthritis Rheum..

[8] Volkmann E.R., Varga J. (2019). Emerging targets of disease-modifying therapy for systemic sclerosis. Nat. Rev. Rheumatol..

[9] Chung M.P., Chung L. (2020). Drugs in phase I and phase II clinical trials for systemic sclerosis. Expert Opin. Investig. Drugs.

[10] Ebata S., Yoshizaki A., Oba K., Kashiwabara K., Ueda K., Uemura Y., Watadani T., Fukasawa T., Miura S., Yoshizaki-Ogawa A. (2021). Safety and efficacy of rituximab in systemic sclerosis (DESIRES): A double-blind, investigator-initiated, randomized, placebo-controlled trial. Lancet Rheum..

[11] Goswami R.P., Ray A., Chatterjee M., Mukherjee A., Sircar G., Ghosh P. (2021). Rituximab in the treatment of systemic sclerosis-related interstitial lung disease: A systematic review and meta-analysis. Rheumatology.

[12] Nakamura K., Yoshizaki A., Takahashi T., Saigusa R., Taniguchi T., Asano Y., Gonoi W., Hinata M., Shinozaki-Ushiku A., Sato S. (2016). The first case report of fatal acute pulmonary dysfunction in a systemic sclerosis patient treated with rituximab. Scand. J. Rheumatol..

[13] Richmond A., Thomas H.G. (1986). Purification of melanoma growth stimulatory activity. J. Cell. Physiol..

[14] Bechara C., Chai H., Lin P.H., Yao Q., Chen C. (2007). Growth-related oncogene-alpha (GRO-alpha): Roles in atherosclerosis, angiogenesis, and other inflammatory conditions. Med. Sci. Monit..

[15] Zeng Y., Lin Q., Yu L., Wang X., Lin Y., Zhang Y., Yan S., Lu X., Li Y., Li W. (2021). Chemokine CXCL1 as a potential marker of disease activity in systemic lupus erythematosus. BMC Immunol..

[16] Furuse S., Fujii H., Kaburagi Y., Fujimoto M., Hasegawa M., Takehara K., Sato S. (2003). Serum concentrations of the CXC chemokines interleukin 8 and growth-regulated oncogene-alpha are elevated in patients with systemic sclerosis. J. Rheumatol..

[17] Van Den Hoogen F., Khanna D., Fransen J., Johnson S.R., Baron M., Tyndall A., Matucci-Cerinic M., Naden R.P., Medsger T.A., Carreira P.E. (2013). 2013 classification criteria for systemic sclerosis: An American college of rheumatology/European league against rheumatism collaborative initiative. Arthritis Rheumatol..

[18] Khanna D., Furst D.E., Clements P.J., Allanore Y., Baron M., Czirjak L., Distler O., Foeldvari I., Kuwana M., Matucci-Cerinic M. (2017). Standardization of the modified Rodnan skin score for use in clinical trials of systemic sclerosis. J. Scleroderma Relat. Disord..

[19] Muangchan C., Harding S., Khimdas S., Bonner A., Baron M., Pope J., Canadian Scleroderma Research Group (2012). Association of C-reactive protein with high disease activity in systemic sclerosis: Results from the Canadian Scleroderma Research Group. Arthritis Care Res..

[20] Korbecki J., Barczak K., Gutowska I., Chlubek D., Baranowska-Bosiacka I. (2022). CXCL1: Gene, Promoter, Regulation of Expression, mRNA Stability, Regulation of Activity in the Intercellular Space. Int. J. Mol. Sci..

[21] Taniuchi N., Ghazizadeh M., Enomoto T., Matsuda K., Sato M., Takizawa Y., Jin E., Egawa S., Azuma A., Gemma A. (2009). Evaluation of fractional analysis of bronchoalveolar lavage combined with cellular morphological features. Int. J. Med. Sci..

[22] Kase K., Watanabe S., Saeki K., Waseda Y., Takato H., Ichikawa Y., Murata A., Yasui M., Noriyuki O., Hara J. (2021). Fractional analysis of bronchoalveolar lavage in systemic sclerosis-associated interstitial lung disease. J. Thorac. Dis..

[23] Liang M., Jiang Z., Huang Q., Liu L., Xue Y., Zhu X., Yu Y., Wan W., Yang H., Zou H. (2016). Clinical Association of Chemokine (C-X-C motif) Ligand 1 (CXCL1) with Interstitial Pneumonia with Autoimmune Features (IPAF). Sci. Rep..

[24] Shattuck R.L., Wood L.D., Jaffe G.J., Richmond A. (1994). MGSA/GRO transcription is differentially regulated in normal retinal pigment epithelial and melanoma cells. Mol. Cell. Biol..

[25] Cuenca R.E., Azizkhan R.G., Haskill S. (1992). Characterization of GRO alpha, beta and gamma expression in human colonic tumors: Potential significance of cytokine involvement. Surg. Oncol..

[26] Huang F., Kao C.Y., Wachi S., Thai P., Ryu J., Wu R. (2007). Requirement for both JAK-mediated PI3K signaling and ACT1/TRAF6/TAK1-dependent NF-kappaB activation by IL-17A in enhancing cytokine expression in human airway epithelial cells. J. Immunol..

[27] You Z., Ge D., Liu S., Zhang Q., Borowsky A.D., Melamed J. (2012). Interleukin-17 Induces Expression of Chemokines and Cytokines in Prostatic Epithelial Cells but Does Not Stimulate Cell Growth In Vitro. Int. J. Med. Biol. Front..

[28] Wu H.H., Hwang-Verslues W.W., Lee W.H., Huang C.K., Wei P.C., Chen C.L., Shew J.Y., Lee E.Y., Jeng Y.M., Tien Y.W. (2015). Targeting IL-17B-IL-17RB signaling with an anti-IL-17RB antibody blocks pancreatic cancer metastasis by silencing multiple chemokines. J. Exp. Med..

[29] Lisi S., Sisto M., Lofrumento D.D., D’Amore M., De Lucro R., Ribatti D. (2013). A potential role of the GRO-α/CXCR2 system in Sjögren’s syndrome: Regulatory effects of pro-inflammatory cytokines. Histochem. Cell Biol..

[30] Scala E., Pallotta S., Frezzolini A., Abeni D., Barbieri C., Sampogna F., De Pita O., Puddu P., Paganelli R., Russo G. (2004). Cytokine and chemokine levels in systemic sclerosis: Relationship with cutaneous and internal organ involvement. Clin. Exp. Immunol..

[31] Hasegawa M., Sato S., Fujimoto M., Ihn H., Kikuchi K., Takehara K. (1998). Serum levels of Interleukin 6 (IL-6), oncostatin M, soluble IL-6 receptor, and soluble gp130 in patients with systemic sclerosis. J. Rheumatol..

[32] De Santis M., Bosello S., La Torre G., Capuano A., Tolusso B., Pagliari G., Pistelli R., Maria Danza F., Zoli A., Ferraccioli G. (2005). Functional, radiological and biological markers of alveolitis and infections of the lower respiratory tract in patients with systemic sclerosis. Respir. Res..

[33] Koch A.E., Kronfeld-Harrington L.B., Szekanecz Z., Cho M.M., Haines K., Harlow L.A., Strieter R.M., Kunkel S.L., Massa M.C., Barr W.G. (1993). In situ expression of cytokines and cellular adhesion molecules in the skin of patients with systemic sclerosis. Their role in early and late disease. Pathobiology.

[34] Kadono T., Kikuchi K., Ihn H., Takehara K., Tamaki K. (1998). Increased production of interleukin 6 and interleukin 8 in scleroderma fibroblasts. J. Rheumatol..

[35] Kondo K., Okada T., Matsui T., Kato S., Date K., Yoshihara M., Nagata Y., Takagi H., Yoneda M., Sugie I. (2001). Establishment and characterization of a human B cell line from the lung tissue of a patient with scleroderma; extraordinary high level of IL-6 secretion by stimulated fibroblasts. Cytokine.

[36] Matsushita T., Hasegawa M., Yanaba K., Kodera M., Takehara K., Sato S. (2006). Elevated serum BAFF levels in patients with systemic sclerosis: Enhanced BAFF signaling in systemic sclerosis B lymphocytes. Arthritis Rheum..

[37] Matsushita T., Fujimoto M., Hasegawa M., Matsushita Y., Komura K., Ogawa F., Watanabe R., Takehara K., Sato S. (2007). BAFF antagonist attenuates the development of skin fibrosis in tight-skin mice. J. Investig. Dermatol..

[38] Bosello S., De Santis M., Lama G., Spanò C., Angelucci C., Tolusso B., Sica G., Ferraccioli G. (2010). B cell depletion in diffuse progressive systemic sclerosis: Safety, skin score modification and IL-6 modulation in an up to thirty-six months follow-up open-label trial. Arthritis Res. Ther..

[39] Tanasescu C., Balanescu E., Balanescu P., Olteanu R., Badea C., Grancea C., Vagu C., Bleotu C., Ardeleanu C., Georgescu A. (2010). IL-17 in cutaneous lupus erythematosus. Eur. J. Intern. Med..

[40] Zhang L., Li Y.G., Li Y.H., Qi L., Liu X.G., Yuan C.Z., Hu N.W., Ma D.X., Li Z.F., Yang Q. (2012). Increased frequencies of Th22 cells as well as Th17 cells in the peripheral blood of patients with ankylosing spondylitis and rheumatoid arthritis. PLoS ONE.

[41] Blauvelt A. (2008). T-helper 17 cells in psoriatic plaques and additional genetic links between IL-23 and psoriasis. J. Investig. Dermatol..

[42] Murata M., Fujimoto M., Matsushita T., Hamaguchi Y., Hasegawa M., Takehara K., Komura K., Sato S. (2008). Clinical association of serum interleukin-17 levels in systemic sclerosis: Is systemic sclerosis a Th17 disease?. J. Dermatol. Sci..

[43] Okamoto Y., Hasegawa M., Matsushita T., Hamaguchi Y., Huu D.L., Iwakura Y., Fujimoto M., Takehara K. (2012). Potential roles of interleukin-17A in the development of skin fibrosis in mice. Arthritis Rheum..

